# Next Generation of Voluntary PRRS Virus Regional Control Programs

**DOI:** 10.3389/fvets.2021.769312

**Published:** 2021-11-05

**Authors:** Edison S. Magalhães, Jeffrey J. Zimmerman, Derald J. Holtkamp, Dyneah M. Classen, Douglas D. Groth, Lauren Glowzenski, Reid Philips, Gustavo S. Silva, Daniel C. L. Linhares

**Affiliations:** ^1^Department of Veterinary Diagnostic and Production Animal Medicine, College of Veterinary Medicine, Iowa State University, Ames, IA, United States; ^2^Carthage Veterinary Service, Ltd., Carthage, IL, United States; ^3^TriOak Foods Inc., Oakville, IA, United States; ^4^Boehringer Ingelheim Animal Health USA Inc., Atlanta, GA, United States

**Keywords:** PRRS (porcine reproductive and respiratory syndrome virus), swine, regional, monitoring, elimination

## Abstract

Porcine reproductive and respiratory syndrome virus (PRRSV) became pandemic in the 1980's and today remains one of the most significant pathogens of the global swine industry. At the herd level, control of PRRSV is complicated by its extreme genetic diversity and its ability to persist in pigs, despite an active immune response. Ultimately, PRRSV control or elimination requires the coordination and active cooperation of producers and veterinarians at the regional level. Early voluntary PRRSV regional control programs focused on routine diagnostic testing and voluntary data-sharing regarding the PRRSV status of participants' herds, but no pre-defined action plans or decision trees were developed to secure project successes (or recover from failures). Given that control of PRRSV is paramount to producer profitability, we propose a coordinated approach for detecting, controlling, and ultimately eliminating wild-type PRRSV from herds participating in regional projects. Fundamental to project success is real-time, multi-platform communication of all data, information, and events that concern the regional project and project participants. New to this approach is the concept of agreed-upon action plans to be implemented by project participants in response to specific events or situations. The simultaneous and coordinated implementation of these strategies allows for early detection of wild-type PRRSV virus introductions and rapid intervention based on agreed-upon response plans. An example is given of a project in progress in the Midwest USA.

## Introduction

Porcine reproductive and respiratory syndrome virus (PRRSV) is an RNA virus that was first recognized in the late 1980's and is now endemic to commercial swine populations throughout the world (1), divided in types I (European) and II (North American) genotypes. The economic impact of PRRSV is significant, e.g., $664 million annually in the U.S. (2) and, at the herd level, €126 per sow in Dutch sow herds undergoing PRRSV outbreaks (3). At the herd level, control of PRRSV is complicated by the virus' genetic diversity and its ability to persist in pigs for weeks or months (4). At the regional level, PRRSV control requires the cooperation of neighboring producers and veterinarians dedicated to stopping area spread (5).

The first voluntary PRRSV regional control program in the USA (2003) was the result of producers' acknowledgment of the economic impact of PRRSV and the recognition that PRRSV-infected herds present a risk to the negative herds in the region (6). In the U.S., the initial producer-driven regional control programs were based on routine diagnostic testing for PRRSV and voluntary data-sharing regarding virus circulation in participants' herds, with the expectation that data sharing *per se* would enhance control (7, 8). To some extent, this expectation was fulfilled. For example, analysis of the Minnesota N212 regional control project showed that a reduction in the incidence of PRRS was associated with a higher degree of voluntary compliance with project-recommended practices (7). On the other hand, sole reliance on PRRSV control through monitoring is generally insufficient; a pre-defined action plan needs to be ready to be triggered in the event of specific circumstances, e.g., outbreaks in participant herds, to maintain momentum and ensure the progress on disease control over time.

Past efforts as described by Wright et al. (8) have shown that a coordinated approach based on defined monitoring protocols, a clear scheme of herd and region PRRSV classification, and pre-agreed action plans are essential in regional PRRSV control. On the other hand, Wright et al. (8) described that the voluntary characteristic of previous regional projects also leads to many pitfalls that impacts it survivability, differently from mandatory programs for example that guarantee the implementation of action plans and full adherence of participants. In addition, the site status in voluntary projects is not updated regularly, which leads to imprecise regional status, and delayed interventions (8).

Despite of these limitations, Rathkjen et al. (9) described that clearly defined PRRSV vaccination protocols, combined with pig flow tailored to farm-specific PRRSV conditions, and biosecurity compliance are effective strategies to eliminate the virus in a region. Also, recent improvements in biosecurity, disease management, and PRRSV monitoring present new opportunities to boost voluntary regional control efforts (1). Therefore, the objective of this study was to develop guidelines for future PRRSV RCPs, by addressing issues from past regional control projects with the technical developments and their implementation.

## Guidelines for Future PRRSV Regional Control Programs (RCPs)

The first step in a regional disease control project is to clearly define the short and long-term goals and the metrics by which progress will be measured, e.g., a reduction in wild-type PRRSV incidence and prevalence within the region, reduction in the genetic diversity of wild-type PRRSV variants in the region, and improvements in whole-herd performance, e.g., pigs weaned per sow per year (PWSY), wean-to-finish (W2F) mortality, and average daily weight gain (ADG). The long-term goal may be to sustain the short-term goals and to fully eradicate wild-type PRRSV from the selected region.

Strategies for fulfilling project goals are based on the following:

1. Participation of the producers in the development of all protocols and procedures.
2. Well-defined PRRSV diagnostic monitoring protocols linked to regional and site-specific PRRSV status classification;
3. At regional and herd levels, ongoing and automated integration of PRRSV diagnostic information with productivity and health data.
4. Action procedures for eradicating wild-type virus based on ongoing site and regional wild-type PRRSV classification;
5. Knowledge of the ecology of wild-type PRRSV;

The initial step in PRRSV RCPs is clearly defining the goals of the project and identifying participants committed to the implementation of the project. Mondaca et al. (10) described five steps to this process: (1) assess the feasibility of the project; (2) identify sites with pigs in the project area; (3) characterize PRRSV status of pig sites; (4) design site-level PRRS control strategies; (5) execute and monitor PRRS control strategies.

### PRRSV Monitoring

In breeding herds, one of the goals of monitoring is to detect PRRSV infections. Thus, the monitoring program must be capable of detecting PRRSV-related changes early and rapidly triggering the appropriate response measures. At the same time, the monitoring program should be practical and cost-efficient. In the last decade, swine producers and veterinarians have implemented new methods for sow herd and piglet population PRRSV monitoring (11) using “processing fluids” (PF) (12) and “family oral fluids” (FOF) (13). Compared to individual pig-based monitoring, PF and FOF samples improved the probability of PRRSV detection while reducing cost, time, and labor. Efficient monitoring for PRRSV can be achieved by submitting samples for testing (nucleic acid or antibody) to a diagnostic laboratory capable of providing diagnostic results on a real-time basis through a secure (password protected) personal account. In addition, continuous analysis of weekly key performance indicators in breeding herds (i.e., abortions or pre-weaning mortality) using statistical process control (SPC) based methods are practical and highly effective for the early identification of PRRS outbreaks (14).

PRRSV control in growing sites remains a tremendous “opportunity” for improvement. Growing pig sites are a source of virus to other sites and effective regional control requires routinely tracking their PRRSV status. Growing sites have greater viral genetic diversity (15) and a higher risk of positive status (16, 17) when compared to breeding herds. Also, Trevisan et al. (18) reported an increased detection of PRRSV in growing sites preceded increased detection in sow farms. Thus, a coordinated approach between multiple sites and companies within a region is mandatory to PRRSV regional control and elimination.

### PRRSV Classifications for Breeding Herds, Growing Sites, and Regions

A well-defined and standardized system for classifying the PRRSV status of herds participating in RCPs is crucial. Essentially, herd classification provides a road map for farms seeking control and/or elimination of PRRSV. In all cases, the goal should be to produce wild-type PRRSV-negative pigs in 8 to 10 months using specific control practices, e.g., stimulation of population PRRSV immunity via exposure to live virus inoculation (LVI) or modified-live virus (MLV) vaccination, herd closure (interrupt the introduction of gilts), McRebel™ (19), and other procedures described in detail elsewhere (6, 13).

#### Breeding Site Classification

Holtkamp et al. (20) originally proposed a breeding herd classification system that was widely used and has been recently updated (21). As in the original classification system, the updated classifications are based on serum testing of weaned piglets, with the addition of population-based methods like family oral fluids or processing fluids. In the present paper, we summarize the new classification system proposed by Holtkamp et al. (21), which is available in the literature for more details, and should be used in conjunction with PRRSV monitoring based on serum and processing fluid (PF) testing in breeding herds, and oral fluids in growing sites. These population-based monitoring methods are practical, accurate, and easy to implement by farm staff (12, 22–25).

- **Site I-A. Positive unstable, high prevalence**. The positive unstable (I-A) status represents breeding herds with epidemic PRRSV circulation. Typically, PRRSV RNA can be detected in serum and/or processing fluid samples and there are PRRSV-associated clinical signs. In addition, herds for which no diagnostic evaluation has been performed are classified as I-A until shown to be otherwise.
- **Site I-B. Positive unstable, low prevalence**. Promotion to status I-B is based on evidence of solid herd immunity and low wild-type PRRSV activity in the herd. This can be achieved by demonstrating at least 10 weekly PCR-negative processing fluids results from 1 week age piglets, and at least 3 of 4 monthly PCR-negative results from due-to-wean piglet sera.
- **Site II or IIvx. Positive stable**. After achieving I-B status, the monthly serum collection in weaning-age pigs is increased to 60 animals (pooled by 10 for testing). Closed herds move from status I-B to status II or IIvx after 13 consecutive weeks of RT-PCR negative processing fluid samplings and 4 consecutive months of negative due-to-wean serum samplings. Regardless of negative sampling results over time, it should be understood that PRRSV may still present in the herd at near-zero prevalence. Thus, surveillance should be continued with monthly RT-PCR testing in weaning-age piglets (serum from 30 pigs) to provide evidence of on-going stable status.
  When herd closure is as part of the PRRSV stabilization program, it is common to begin to introduce replacement gilts into the sow herd when herds achieve II or IIvx status.
  When the control plan is to remain in status IIvx through periodic sow herd mass MLV vaccination, incoming gilts should be immunized at least 2 months prior to introduction to the breeding herd, ensuring that they have prior immunity and are non-shedding (1). In IIvx herds, any unexpected PRRSV RT-PCR positive results should be further characterized (i.e., open reading frame (ORF)-5 or whole-genome sequencing) to rule out circulation of wild-type PRRSV.
- **Site III. Provisional negative**. Status III is a transitional status for herds on the path to eliminate the virus and therefore no PRRSV vaccination or LVI is implemented at this stage. Status III is achieved by demonstrating that 60 “sentinel” replacement gilts were PRRSV antibody-negative 60 days after introduction and by documenting that, since gilt introduction, the sow herd has continued to produce PRRSV-free piglets, as evidenced by 8 consecutive weeks of PRRSV RT-PCR-negative processing fluids and 2 consecutive negative monthly weaned pig serum samples.
- **Site IV. Negative**. Status IV denotes herds naïve for the virus. Status IV is achieved by demonstrating that sows (*n* = 60) are PRRSV antibody-negative and piglets are free of PRRSV infection via on-going processing fluid and weaned pig serum testing.

### Growing Site Classification

Establishing the status of growing sites (negative, vaccine virus positive, or wild-type PRRSV positive) is important because sites infected with wild-type virus pose a threat to the success of regional disease control projects. Status can be determined based on monthly oral fluid sampling (6 samples) and testing by PRRSV RT-PCR followed by sequencing of unexpected positives to differentiate wild-type virus from vaccine virus. In expected naïve sites, PRRSV oral fluid antibody ELISA can be used to detect infection. To increase the probability of detecting the virus at low prevalence scenario, collect oral fluid samples from pens spaced equidistantly from each other (i.e., spatial sampling) within rooms or barns and sample the same pens over time.

#### Growing Site Status

Using this protocol, growing site status is defined by testing outcome.

- **Positive growing sites** are defined by ≥ 1 PRRSV RT-PCR or ELISA positive oral fluid sample(s) from which a wild-type PRRSV sequence is subsequently obtained. Wild-type virus may have originated from the sow herd, e.g., sow herds in status I-A or I-B or may have been introduced from another source after placement.
- **Vaccinated growing sites** are defined by ≥ 1 PRRSV RT-PCR positive oral fluid sample(s) from which there is no evidence of wild-type PRRSV circulation (i.e., a vaccine-like sequence is obtained). Vaccinated growing sites are typically sourced from status II or II-vx sow herds.
- **Seropositive, non-shedding growing sites** are defined by PRRSV RT-PCR-negative oral fluid samples that are ELISA positive, when tested. These groups typically originate from status II, IIvx, or III sow herds.
- **Negative growing sites** are defined by continued negative PRRSV RT-PCR and ELISA oral fluid testing results over time. These groups typically originate from status IV sow herds.

### PRRSV Regional Classification

Regional classification is an aggregate measure based on the PRRSV status of sites within the region (Table 1). Its purpose is to measure project progress over time and inform herd-level action plans. For example, when a region is positive at high prevalence, extreme herd-level PRRSV control measures to achieve naïve status, e.g., depopulation, would be unreasonable because of the high probability of reinfection. Alternatively, if the regional status is infected at low prevalence, more aggressive herd-level control efforts may be justified to prevent other herds to become infected. Thus, regional classification serves as a guide for production systems in terms of making decisions that will avoid PRRSV losses and eventually lead to PRRSV elimination.

**Table 1:** Regional classification based on prevalence of wild-type PRRSV unstable sites.

**Regional classification**	Criterion^*^
Status I-H (infected, high prevalence)	>50% sites unstable with wild-type PRRSV
Status I-M (infected, moderate prevalence)	20–50% sites unstable with wild-type PRRSV
Status I-L (infected, low prevalence)	≤ 20% sites unstable with wild-type PRRSV
Status P-N (provisional negative)	No wild-type PRRSV detected for <6 months
Status N (negative)	No wild-type PRRSV detected for ≥6 months

* *Regional wild-type PRRSV prevalence calculated as (number of unstable sites ÷ total number of sites). Unstable sites include Status I-A breeding herds, positive growing sites, and any site of unknown status*.

### PRRSV Control Protocols

The simultaneous implementation of immunization and biosecurity strategies is used to control PRRSV in RCPs. The goal of herd immunization is to ameliorate production losses, reduce cyclic outbreaks produced by PRRS viruses endemic to the herd (26–29), and produce wild-type PRRSV-negative piglets within 8–10 months. For biosecurity, the goal is to reduce the frequency of outbreaks with unrelated PRRSV strains by reducing the likelihood of the introduction of the virus (bioexclusion) and, on endemically-infected farms, limit the dissemination of virus within a farm (biomanagement). Further, preventing the spread of wild-type virus between herds (biocontainment) is a key component in this set of actions, especially for preventing virus dissemination from growing sites to breeding herds, as mentioned previously.

The main objective of a well-established and agreed-upon regional contingency plan is to implement coordinated control strategies on all sites, as was previously agreed upon by project participants. The actions are focused on positive wild-type PRRSV sites, becoming progressively more aggressive as the regional wild-type PRRSV prevalence decreases (Table 2).

**Table 2:** Guidelines for control protocols in wild-type PRRSV-positive herds within regional status.

**Region**	**Action**	**Breeding herds**	**Growing pig sites**
I-H	Detection	• Herd I-A 10–12 weeks after mass exposure initiate monitor with processing fluids (weekly) and serum (monthly)	• Monthly sampling of groups from placement to marketing
	Management	• Herd closure • Whole-herd exposure and/or routine immunization • Incoming gilts negative for wild-type PRRSV • Gilt acclimation	• MLV vaccination within 40 days of weaning in unvaccinated populations originating from I-A breeding herds • Strict all in-all out by barn. Clean, disinfect between groups • Control people, pig, and vehicle flow on to the production site
I-M	Detection	• Herds II, II-vx, III, and IV re-instate weekly processing fluid monitoring if I-A breeding herds or positive growing sites are located within a 3-mile radius	• Detection as per I-H.
	Management	• Management as per I-H	• As per I-H. • Do not place wild-type PRRSV-positive pigs in growing pig sites
I-L	Detection	• As per I-M.	• As per I-H
	Management	• As per I-M	• Segregate people and vehicles from unstable sites • Double-dose MLV vaccination in wild-type positive groups, if detected ≤ 40 days of weaning (2nd dose 3–4 weeks after 1st dose). If double-dose protocol is not possible, guarantee at least one dose in wild-type PRRSV-positive nursery pigs • One dose MLV vaccination in wild-type PRRSV-negative groups within 3-miles of a wild-type positive site and ≤ 40 days of placement • Alternative to vaccination is depopulation or trade-off of positive groups
P-N/N	Detection and Management	• Detection as per I-M; management as per I-H	• As described for status I-L

### Productivity Data Analysis and Interpretation

On-going analysis of breeding herd productivity data using statistical process control (SPC) techniques will often reveal the introduction of wild-type PRRSV into the herd prior to the observation of overt clinical signs. Parameters of interest include a daily count of sows off feed and weekly averages for birth losses (total born – born alive), pre-weaning mortality, and abortions. Ideally, this process would automatically integrate the sow farm electronic data with SPC software thereby assuring real-time alerts.

### Communication, Management, and Leadership

Communication serves to maintain participant awareness and interest in their regional control project and inform regarding events that may affect participants' production strategies and/or decisions. Without active communication, producers quickly lose interest and the project dies for lack of forward momentum. Thus, open and fully transparent communication is a key component in regional control programs and participating production systems should agree to share information.

Maintaining clear, timely, effective, on-going communication is a key component in regional control approaches (10, 30), and is the responsibility of the project coordinator. The responsibility of the project coordinator is to manage all the information collected in the farms, synthesize and interpret on-going diagnostic and SPC data and then communicate updates to project participants. The most critical information concerns the communication of events that directly affect the project and overall regional status, e.g., outbreaks, changes in pig-flow, and changes in production site status. Of particular importance to producers are changes in PRRSV circulation within the project area. Lastly, the project coordinator is the leadership in the project regarding quality assurance of the information, guaranteeing that the schedule of the project is being followed, and summarizing all the information collected into straight forward update reports for the project participants. These reports should be sent weekly, but communication of critical events, e.g., the new introduction of wild-type PRRSV in the region, should occur immediately. To achieve this aim, the project coordinator should use communication channels available as needed, e.g., e-mail, text messaging, digital platforms, and develop new approaches where needed. Ideally, communications include a “snapshot” of the region, with the identification of the sites and their respective classification.

## Case Study

The concepts and protocols described above reflect the protocols and procedures developed and implemented by participants in an on-going PRRSV regional control project in the Midwest USA. The short-term goals of the project are to achieve regional control of wild-type PRRSV circulation and improve herd performance metrics in both breeding herds and growing pig populations. The long-term project goal is elimination of the wild-type virus from the region. Weekly reports sent to project participants via text messages described changes in site status, an updated sampling schedule, diagnostic results in progress, a map of PRRSV site status, and other pertinent information related to the project. Expenses related to the project were borne by the producers, with monitoring of growing sites subsidized by a third-party company.

Initiated in April 2019, the project had 6 breeding sites (~30,400 sows) and 14 growing pig sites (~50,000 head), i.e., all swine production sites in an area of ~701 km^2^. Project participants monitor sites as previously described in this manuscript and all diagnostic specimens were sent to a veterinary diagnostic laboratory capable of processing the samples and electronically providing results within 24 h upon arrival. In growing sites, monthly monitoring using oral fluids revealed the introduction of wild-type PRRSV in 2 growing sites and confirmed PRRSV “leaking” from sow farms in 4 other sites. In the event of a PRRSV-positive result, the laboratory's software is linked to a visualization and mapping platform, and the site classification is immediately updated to the new infected status, once the site status is changed in the VDL platform when accessing the diagnostic information and other relevant information (clinical signs) to confirm this modification. For the purpose of this study the “Disease BioPortal^®^” (https://bioportal.ucdavis.edu) platform was utilized, however any other geographical business intelligence tools can be utilized, for example Tableau^®^ (Tableau Software, Seattle, WA) or Power BI^®^ (Microsoft Corporation, Redmond, Washington). If the automated communication between the VDL and visualization software is not available, the project coordinator is responsible to gather incoming diagnostic results and other relevant data, and use it to manually update the status of the region in the visualization software, as seen that the final goal is to provide weekly visual “snapshots” of the region wild-type PRRSV prevalence.

Breeding herds in the project perform web-based SPC monitoring of sows off-feed, birth losses, pre-weaning mortality, and abortions on a weekly basis (https://fieldepi.cvm.iastate.edu/fieldepi), with sigma (σ) and smoothing parameter (λ) values set at 3 and 0.40, respectively. For each site, the software generates a SPC chart and sends an automated email to alert the participant of a significant deviation, i.e., a value outside the three-sigma limit (14). Since the initiation of the project there have been a total of 64 significant SPC deviations (16 for abortions, 13 for birth losses, and 35 for pre-weaning mortality) in 4 different sow farms. Four of the SPC abortion alerts led to the detection of wild-type PRRSV in 3 breeding herds, as confirmed by PRRSV RT-PCR positive results on processing fluids and ORF-5 sequencing. Within 4 days of the SPC alerts, the diagnostics had been performed and project participants were informed of the wild-type PRRSV outbreaks in the region.

The coordinated utilization of the previously described tools assisted the project coordinator in managing, interpreting and communicating updates and progress to participants (31). The utilization of platforms available from Veterinary Diagnostic Laboratories (VDL's), along with a robust monitoring protocol for diagnostic testing and productivity parameters, allows for early identification of a wild-type introduction and, the most importantly, the immediate implementation of actions based on the pre-defined PRRSV control protocols. For example, the introduction of wild-type PRRSV into a growing site when the region was below 20% prevalence (Status I-L) resulted in the removal of the PRRSV-positive pigs to a site outside the region as part of the agreed-upon action plan for this scenario. Thus, project participants and especially surrounding sites were protected against exposure to wild-type PRRSV.

## Conclusions/Observations

Disease eradication projects for World Organisation for Animal Health (OIE)-listed diseases are typically driven by government regulations and financial support. For economically impactful endemic diseases, such as PRRSV, producers must take responsibility and act on their own behalf to create and implement programs to control PRRSV and mitigate its economic impact. Experience has shown that the most effective strategy to achieve and preserve this goal is at a regional level. We believe that producer-driven regional control projects based on the routine collection of productivity and surveillance data and using clear rules and guidelines to coordinate actions can successfully achieve PRRSV control and (finally) elimination.

## Data Availability Statement

The original contributions presented in the study are included in the article, further inquiries can be directed to the corresponding author/s.

## Author Contributions

EM wrote the first draft of the manuscript. All authors contributed on the development of the guidelines described in the present paper, contributed to manuscript revision, read, and approved the submitted version.

## Funding

This study was partially funded by Boehringer Ingelheim Animal Health USA Inc. The funder was involved in the writing of this article.

## Conflict of Interest

DC and DG are employed by Carthage Veterinary Service, Ltd. LG is employed by TriOak Foods Inc. RP is employed by Boehringer Ingelheim Animal Health USA Inc. The remaining authors declare that the research was conducted in the absence of any commercial or financial relationships that could be construed as a potential conflict of interest.

## Publisher's Note

All claims expressed in this article are solely those of the authors and do not necessarily represent those of their affiliated organizations, or those of the publisher, the editors and the reviewers. Any product that may be evaluated in this article, or claim that may be made by its manufacturer, is not guaranteed or endorsed by the publisher.
